# Metamaterial Vivaldi Antenna Array for Breast Cancer Detection

**DOI:** 10.3390/s22103945

**Published:** 2022-05-23

**Authors:** Marwa Slimi, Bassem Jmai, Hugo Dinis, Ali Gharsallah, Paulo Mateus Mendes

**Affiliations:** 1 Department of Physics, FST, Microwave Electronics Research Laboratory, University of Tunis El Manar, Tunis 4800058, Tunisia; slimimarwa8@gmail.com (M.S.); ali.gharsallah@fst.utm.tn (A.G.); 2 CMEMS-UMinho, University of Minho, 4800-058 Guimarães, Portugal; bassem.jmaiesti@gmail.com (B.J.); hugodcdinis@gmail.com (H.D.); 3 LABBELS—Associate Laboratory, Braga/Guimarães, Portugal

**Keywords:** tumor detection, metamaterial (MTM), antipodal Vivaldi antenna (AVA), antenna array, breast phantom

## Abstract

The objective of this work is the design and validation of a directional Vivaldi antenna to detect tumor cells’ electromagnetic waves with a frequency of around 5 GHz. The proposed antenna is 33% smaller than a traditional Vivaldi antenna due to the use of metamaterials in its design. It has an excellent return loss of 25 dB at 5 GHz and adequate radiation characteristics as its gain is 6.2 dB at 5 GHz. The unit cell size of the proposed metamaterial is 0.058λ × 0.054λ at the operation frequency of 5 GHz. The proposed antenna was designed and optimized in CST microwave software, and the measured and simulated results were in good agreement. The experimental study demonstrates that an array composed with the presented antennas can detect the existence of tumors in a liquid breast phantom with positional accuracy through the analysis of the minimum amplitude of S_ii_.

## 1. Introduction

**Figure 1:**
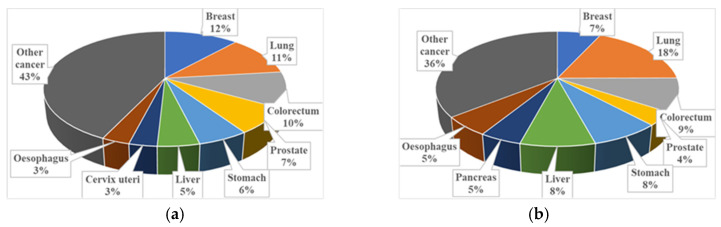
Pie chart showing different types of cancer in 2020 for (**a**) new cases and (**b**) deaths in the world. Data from [1].

According the World Health Organization’s International Agency for Research on Cancer, cancer is the second leading cause of death worldwide, killing approximately 684,996 and affecting 2,261,419 per year. Breast cancer is the most common malignant tumor in women: in 2020, it corresponded to 12% of new cases and 7% of deaths [1], as illustrated in Figure 1.

Research for breast cancer detection is conducted in most countries and different procedures and detection tools are used. The majority of imaging techniques used for breast cancer screening and diagnosis are mammography, echography and magnetic resonance imaging (MRI). The first is fast and has good sensitivity, but it is not comfortable and uses ionizing radiation. Echography is based on ultrasound waves and achieves high resolution, but the diagnostic quality is heavily dependent on the skills of the technician and the expertise of the operator. Finally, MRI provides very high-resolution images, but it is time consuming, expensive and prone to induce patient claustrophobia.

Nowadays, a lot of research is conducted to explore the possibility of detecting tumors in the human body, more particularly in the breast, using microwave signals. The advantage of microwave imaging is that it is a nonionizing and, a priori, inexpensive technique. The antenna is the most important element of a microwave imaging system [2]. Many antennas have been designed for breast cancer detection, such as the horn antenna [3], CPW antenna [4], 3D antenna [5], microstrip antenna [6] and dielectric resonator antenna (DRA) [7]. Many researchers also add different types of metamaterials to improve the characteristics of the antennas for tumor detection. In [8], the authors presented a CPW antenna based on metamaterials with a low cost and ultrawideband (UWB) frequencies (4–15 GHz) used for early breast cancer detection, but this antenna was not directive (omnidirectional antenna) and had a low Q factor and a large size (80 mm × 61 mm). The proposed antenna can detect the presence of tumors but with poor precision of its position since the low Q factor leads to an inadequate signal response, and the low directivity of the antenna does not allow to focus the wave signal towards a precise point. Furthermore, increasing the number of antennas can improve the precision of the system, but the large size of the antenna presented by the authors is a limiting factor.

This paper presents the design, simulation and improvement of a directive metamaterial antipodal Vivaldi antenna (MTM-AVA) for breast cancer detection. It is divided into three parts: the first is dedicated to the design of a classic AVA in the operating frequency; the second presents the miniaturized AVA based on MTM technology. Finally, the experimental results of an antenna array formed using the developed MTM-AVA are presented in the third part.

## 2. Antenna Design

### 2.1. The Proposed Antipodal Vivaldi Antenna (AVA)

**Figure 2:**
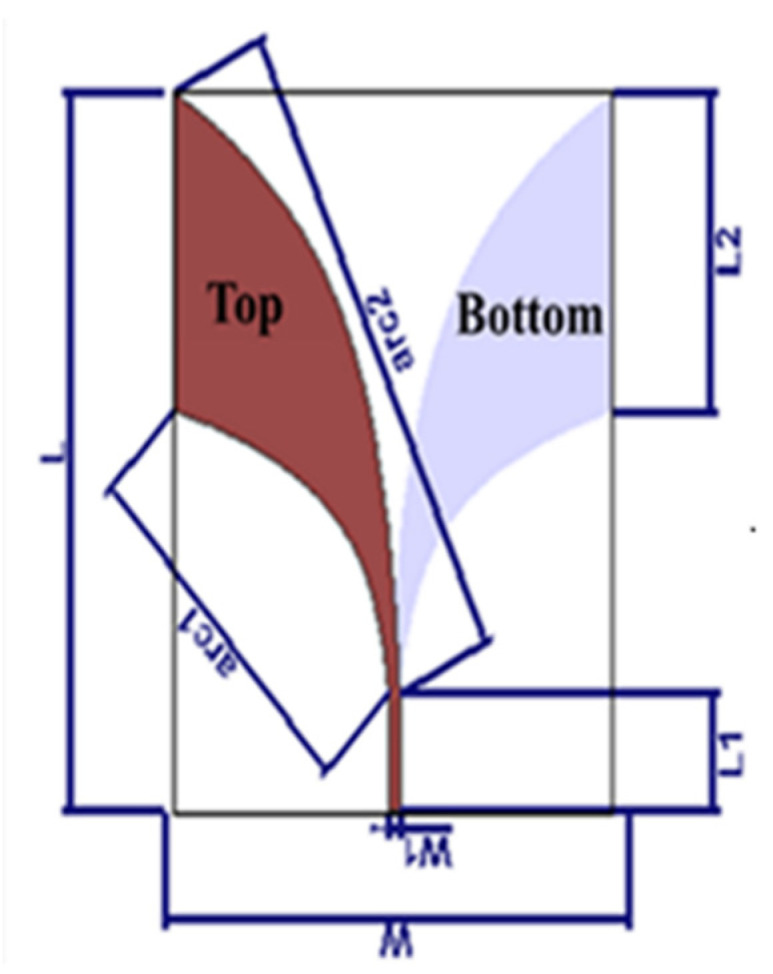
The geometry design of the AVA.

The proposed AVA is shown in Figure 2. It was designed on a Roger substrate (dielectric constant (ε_r_) = 10.2, loss tangent (tanδ) = 0.0022) with a thickness of h = 0.64 mm and copper thickness of t = 0.0175 mm. Table 1 shows the geometrical dimension parameters of the antenna. The antenna is composed of two copper layers. The signal-side copper, which is the top layer, is presented in red and the ‘ground’-side copper, which is the bottom layer, is shown in grey. The feed line of the top layer was adapted to 50 Ω and connected to the RF excitation of the SMA connector while the bottom layer was connected to the SMA connector ground. The taper was designed according to Equations (1) and (2) where *A* = 1.511, m = 0.053352 and n = 0.1118.

(1) $$Arc1=\left\{ \begin{array}{l} x\left(t\right)=t\\ y\left(t\right)=\exp\left(nt\right),t:0\to 30.914-A/4\\ z\left(t\right)=0 \end{array}\right.$$

(2) $$Arc2=\left\{ \begin{array}{l} x\left(t\right)=t\\ y\left(t\right)=-A\exp\left(mt\right),t:0\to 63.38+A\\ z\left(t\right)=0 \end{array}\right.$$

**Figure 3:**
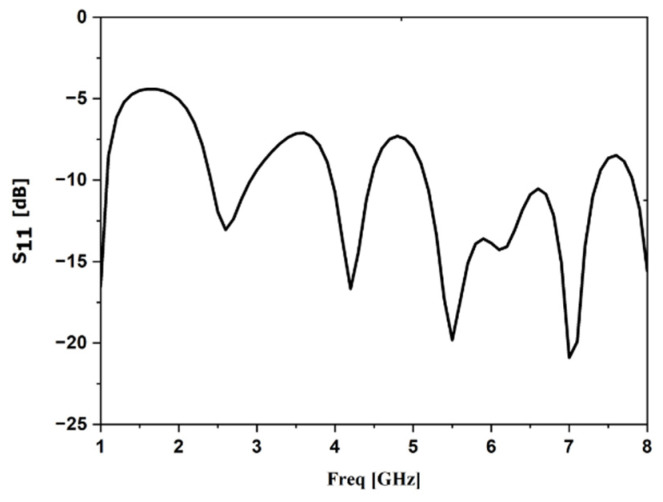
Simulation result of the return loss.

**Figure 4:**
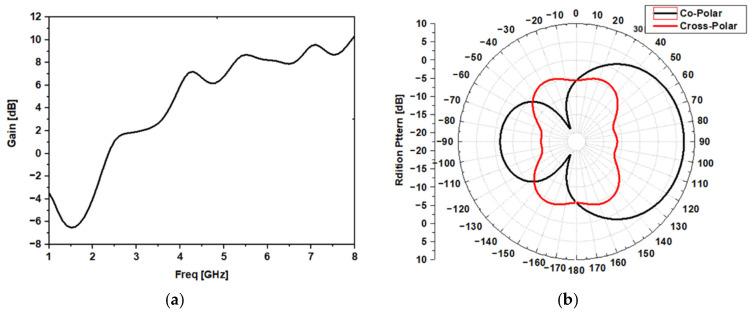
Simulation results of the gain (**a**) versus frequencies (**b**) at 5 GHz.

The design started with a simulation of a standard antipodal Vivaldi using HFSS software: “commercial 3D solver for electromagnetic structures from Ansys based on the finite element method”,. Figure 3 shows the S-parameter simulation results in the free space. Figure 4a shows the gain simulation result versus frequency, and Figure 4b presents the simulated radiation pattern in the proposed frequency for tumor detection (5 GHz), in which it is possible to observe that the proposed antenna is directional with a gain equal to 6 dB.

### 2.2. The Proposed Metamaterial Antipodal Vivaldi Antenna (MTM-AVA)

This section describes the design of the MTM unit and how to improve the characteristics of the antenna to reduce its size, finishing with a presentation of the miniaturized AVA based on the MTM units.

#### 2.2.1. Metamaterial Structure

**Figure 5:**
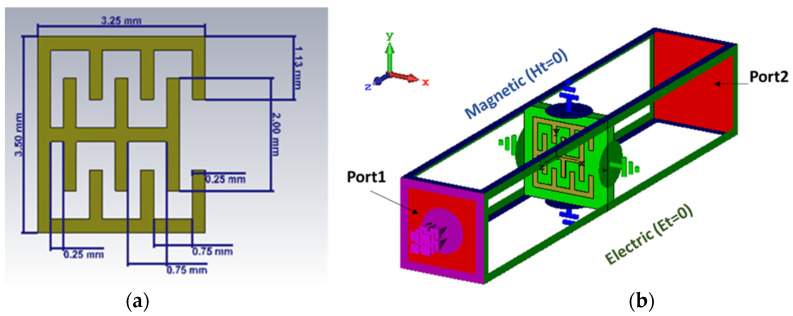
Metamaterial unit cell: (**a**) schematic of the proposed cell model unit; (**b**) 3D view of the simulation setup of the unit cell.

Figure 5a presents the schematic MTM geometry. It consists of a resonator, an E-shape and teeth printed on a Roger 3010 dielectric substrate with a permittivity of 10.2 and 0.64 mm of thickness. Generally, the metamaterials used in the 5 GHz frequency have large dimensions, such as in [9]. The designed resonators are split ring resonators at 2.45, and 5 GHz is 34 × 34 mm^2^, which inspired this model. The unit cell size of the proposed metamaterial is 0.058λ × 0.054λ (3.25 × 3.25 mm^2^) at the operation frequency of 5 GHz. Figure 5b presents the setup simulation box of the proposed MTM using the Computer Simulation Technology-Microwave Studio Suite (CST-MWS) based on the time−domain method. As indicated in the figure, the proposed MTM was placed inside a waveguide with perfect magnetic conductors (PEM) on its top and bottom, perfect electric conductors (PEC) on its two side walls and two waveguide ports for excitation (Port 1 and Port 2).

**Figure 6:**
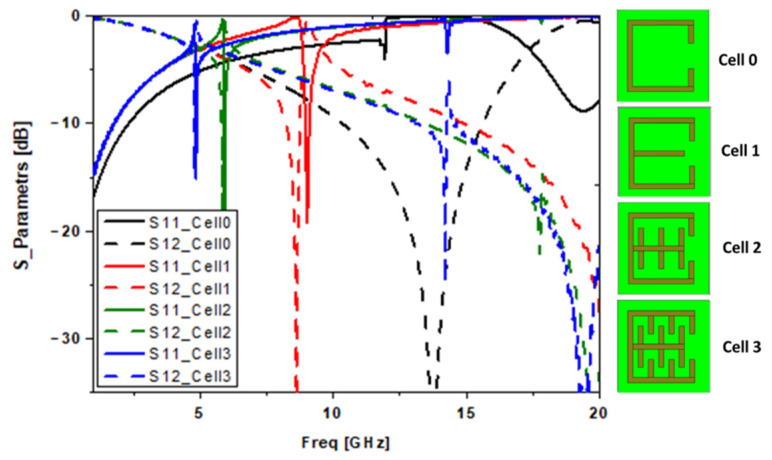
Curve of the reflection and transmission coefficients produced by different proposed structures.

A new E-Shaped slotted resonator with teeth (E-Shaped SRR) is now presented. Starting with the split ring (SRR), which was introduced by Tang Chun Ming in 2010 [10], we added a microstrip line in the middle to obtain an E-shape, which produced a resonance at 9 GHz. Following this, we added teeth (i.e., capacitors and inductors), as shown in Figure 6, to obtain a frequency of 5 GHz.

The basic technical parameters of MTMs are the relative permittivity (*ε*) and the relative permeability (*μ*).

The obtained result of the “recovered method” way can be used to describe the property of the proposed metamaterial. As shown Equations (3) and (4), (*ε*, *μ*) are dependent of the impedance (*z*) and the refractive index (*n*). As such, (*ε*, *μ*) are based on |*S*| parameters since n and Z depend on *S*_11_, *S*_22_, *S*_12_ and *S*_22_ (port 1 and port 2 are presented in Figure 5b), and the substrate thickness (*h*) and the propagation constant (*k*) are provided in Equations (3)–(7) [11,12].

(3) $$\varepsilon =\frac{n}{z}$$

(4) μ=nz

(5) $$z=\sqrt{\frac{{\left(1+S_{11}\right)}^{2}-S_{21}{}^{2}}{{\left(1-S_{11}\right)}^{2}-S_{21}{}^{2}}}$$

(6) $$n=\left(1/kh\right)\cos^{\left(-1\right)}\left[\left(1/2S_{21})(1-S_{11}{}^{2}+S_{21}{}^{2}\right)\right]$$

(7) $$k=\frac{2\pi }{\lambda }$$

**Figure 7:**
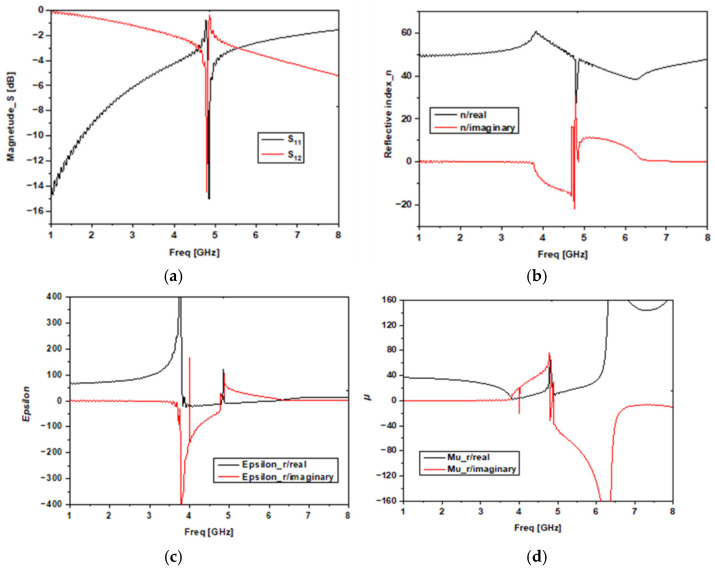
The unit cell results: (**a**) simulated S-parameters, (**b**) refractive index, (**c**) effective permittivity, and (**d**) effective permeability.

The simulated properties of the metamaterial are provided in Figure 7 (Figure 7a shows the *S*-parameters, Figure 7b the refractive index (*n*), Figure 7c the permittivity and Figure 7d the permeability). We can see that in the simulations of the proposed MTM, in the vicinity of 5 GHz, we have the minimum transmission and the maximum reflection and absorption, meaning that it is the resonance frequency.

#### 2.2.2. Antipodal Vivaldi Antenna with Metamaterials: Simulation and Design

**Figure 8:**
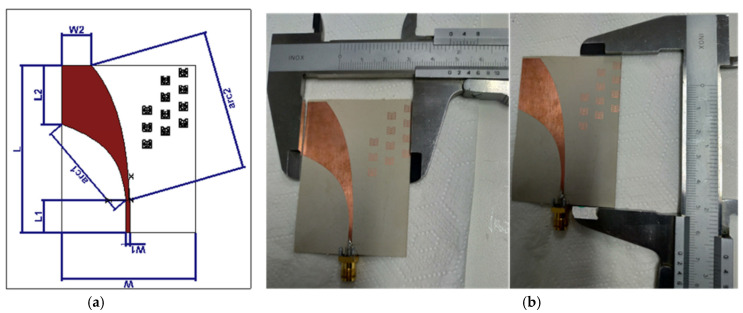
The presented Vivaldi antenna with MTM: (**a**) geometry design (**b**) fabrication and measurements.

The MTM was added to both sides of the antenna substrate to improve the performance of the basic antenna, mainly the adaptation at 5 GHz. We tried several placements and orientations of the proposed metamaterial and concluded that the design shown in Figure 8 produced the best results. Figure 8 shows the modification of the antipodal Vivaldi antenna using loaded MTMs to miniaturize the size and increase the performance. Table 2 summarizes the different structural dimensions of the optimized AVA.

**Figure 9:**
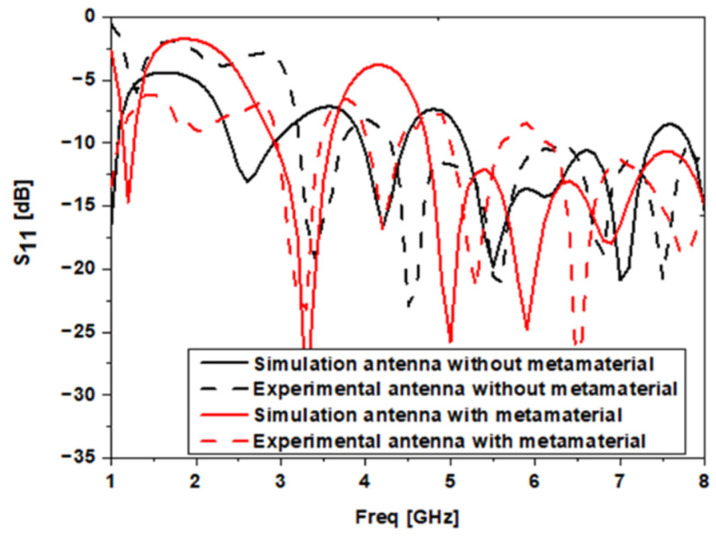
Comparative study of S parameter results of the AVA with and without the metamaterial.

Figure 9 shows the S-parameter results of the modified antenna based on the MTM cells. The antennas had a good performance, the simulation and measurement results were in agreement and the antenna was well matched over the operating frequency bands (based on the measured results, the S_11_ was equal to −26 dB at 5 GHz). We can see in this figure that the metamaterial, besides improving the antenna size reduction, also improves the quality factor [13], which translates into a deeper S_11_ resonance, leading to a better tumor detection.

**Figure 10:**
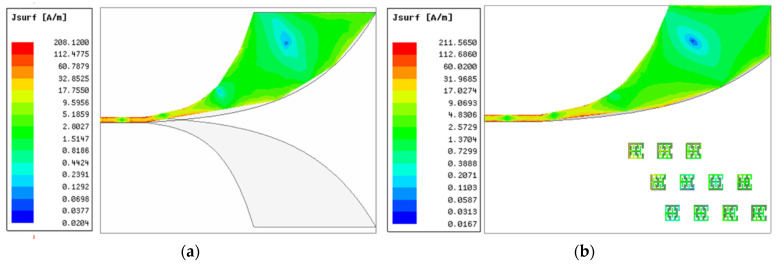
Surface current distribution in the Vivaldi antenna at 5 GHz (**a**) without and (**b**) with the metamaterial.

**Figure 11:**
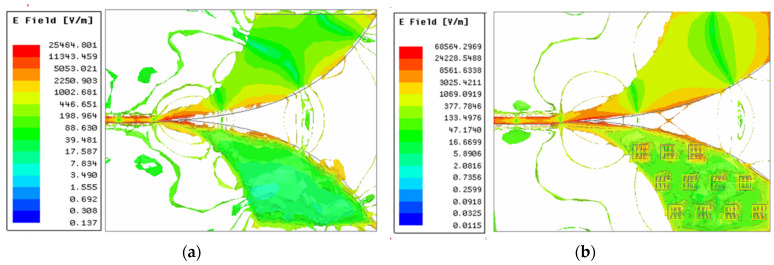
Electric field distribution in the Vivaldi antenna at 5 GHz (**a**) without and (**b**) with the metamaterial.

**Figure 12:**
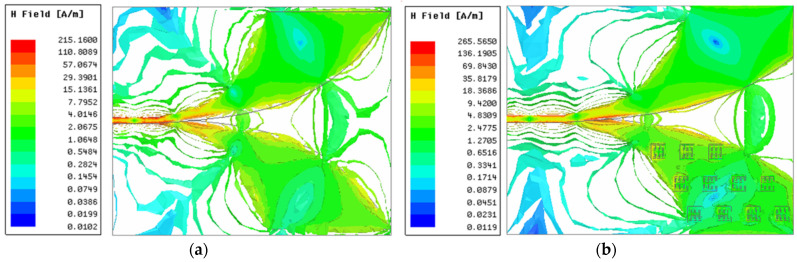
Magnetic field distribution in the Vivaldi antenna at 5 GHz (**a**) without and (**b**) with the metamaterial.

Figure 10, Figure 11 and Figure 12 show, respectively, the simulation result of the peak values in terms of the current distribution, the electric field and the magnetic field in the Vivaldi antenna with and without the metamaterial at 5 GHz. As indicated in Figure 10a, a maximum surface current of 208.12 A/m is generated by the reference Vivaldi antenna at 5 GHz. The proposed MTM-AVA produced 211.57 A/m, which is slightly more, indicating that the MTM contributed to this effect. As indicated in Figure 11 and Figure 12, it is clear that the peak values of the electric and magnetic fields of the Vivaldi antenna with the metamaterial are higher comparative to the peak values of the reference Vivaldi antenna. As such, we can conclude that the proposed metamaterial increases the distribution of electric and magnetic fields.

**Figure 13:**
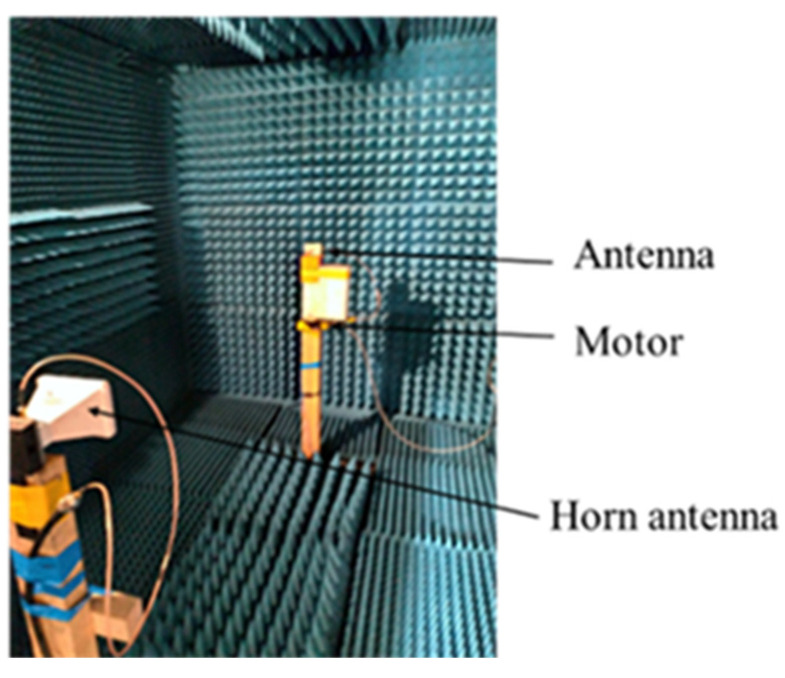
Measurement of the gain in the anechoic chamber.

**Figure 14:**
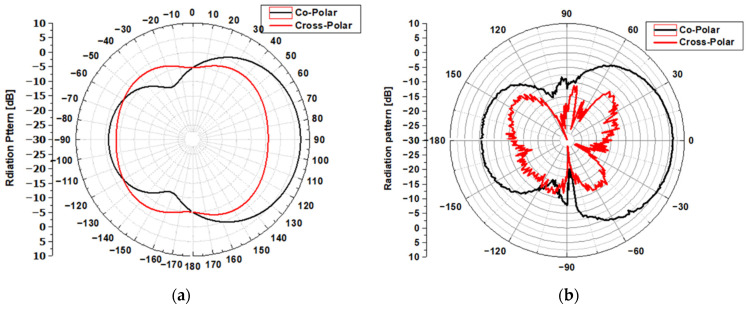
Radiation patterns result in E-plane (phi = 90°) and H-plane (phi = 0°) at 5 GHz in terms of (**a**) simulation results and (**b**) experimental results.

Figure 13 shows the gain measurement setup of the antenna under testing in the anechoic chamber. Figure 14 illustrates the graphic representation of the radiation pattern. The measurement, shown in Figure 14b, confirms the radiation pattern characteristics of the proposed MTM-AVA, as expected from the simulations shown in Figure 14a.

Table 3 shows the comparison of the proposed antenna with the selected existing antenna designs described in the literature. This concludes that the proposed antenna is more appropriate for our system for breast cancer detection and has good directivity for increased precision in tumor detection and localization.

### 2.3. Specific Absorption Rate (SAR) Results

When using radiofrequency signals for biomedical applications, the specific absorption rate (*SAR*), a measure of the energy absorbed per unit mass of tissue (W/kg) by the human body, must be kept under predefined levels to ensure patient safety. The *SAR* can be calculated from the following physical quantities: the electric field *E*, the current density *J*, or the temperature rise *dT/dt* in the tissues, as demonstrated in Equations (8)–(10) [20].

(8) $$SAR(W/\mathrm{kg})=\left(\frac{\sigma {\left|E\right|}^{2}}{2\rho }\right)$$

(9) $$SAR(W/\mathrm{kg})=\left(\frac{{\left|J\right|}^{2}}{\sigma \rho }\right)$$

(10) $$SAR(W/\mathrm{kg})=C_{i}\left(\frac{dT}{dt}\right)$$

**Figure 15:**
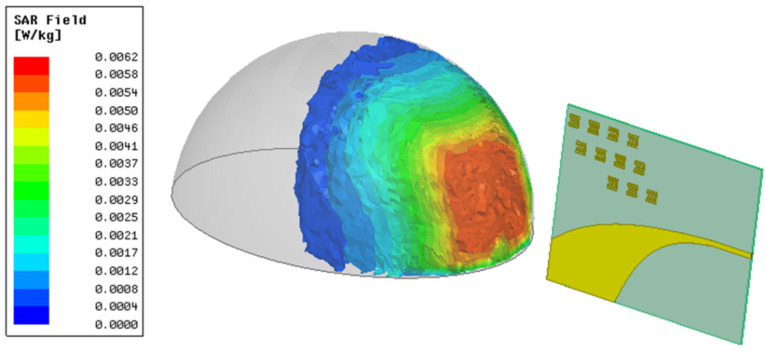
Distribution results of 10 g averaged *SAR* inside the breast phantom at 5 GHz.

Europe defines a *SAR* limit of 2 W/kg averaged over 10 g of tissue for 6 min while the USA sets a slightly stricter limit of 1.6 W/kg over 1 g of tissue for 30 min [21]. Figure 15 shows the *SAR* inside the breast model when it is illuminated by the proposed MTM-AVA, placed 1 cm away from the model and emitting 1 mW of power at 5 GHz.

At an emitted power of 1 mW, the maximum *SAR*_10g_ value on the breast model was calculated to be 6.21 10^–3^ W/kg over 10 g of tissue, as shown in Figure 15. The breast phantom was based on the databases of tissue electrical properties, from the IT’IS Foundation [22], for the different layers. The breast model uses geometric properties of the “model I” from the paper [20]: the skin is 2 mm, the fat is 8 mm, the blood is 2 mm is and the gland is 116 mm.

## 3. Antenna Array for Microwave Breast Cancer Detection

**Figure 16:**
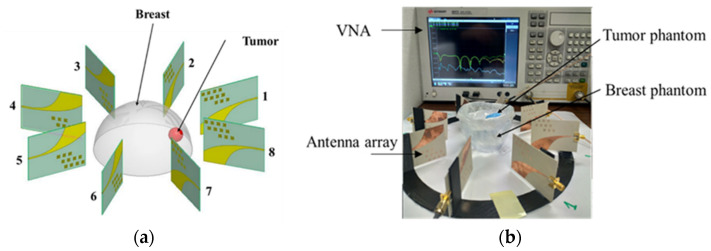
Array antenna for tumor detection: (**a**) conception design of the 8 antennas and the positioning from 1 to 8; (**b**) experimental system.

After antenna and *SAR* characterization, eight antennas were used to form an antenna array to be used as a tumor sensing device. Figure 16 shows the proposed antenna array structure for tumor detection. The antenna array consists of eight elements placed in a circular and symmetrical configuration, and its antennas are staggered by 45°.

**Figure 17:**
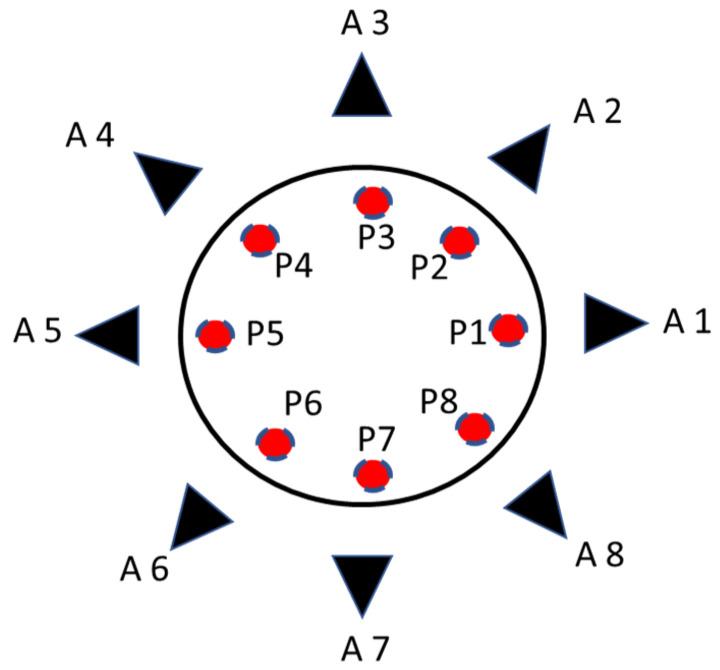
Proposed scenario of the antenna array for tumor detection (antennas: A1 to A8 and the tumor position P1 to P8).

The greatest challenge of an antenna array system is the maximum distance at which the antenna reader can detect the tumor. The eight antennas are placed 25 mm away from the breast phantom’s surface to detect tumor cells. The proposed scenario of the breast cancer detection corresponds to the use of the first antenna as a transmitter and the other seven antennas as receivers. The proposed scenario is shown in Figure 17. In turns, the tumor is placed in positions P1 to P8, and the S-parameters are measured.

**Figure 18:**
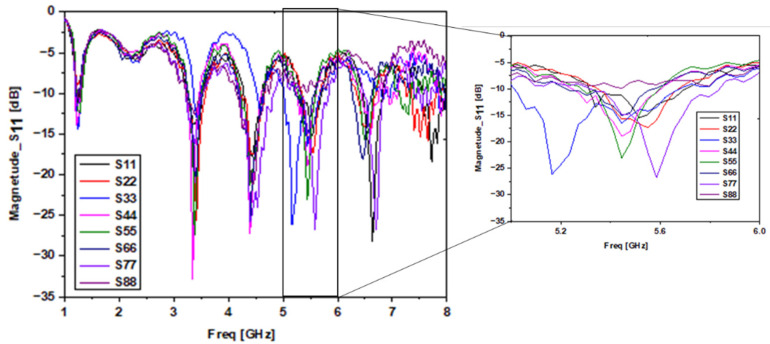
Experimental results of S_ii_ in position 1.

**Figure 19:**
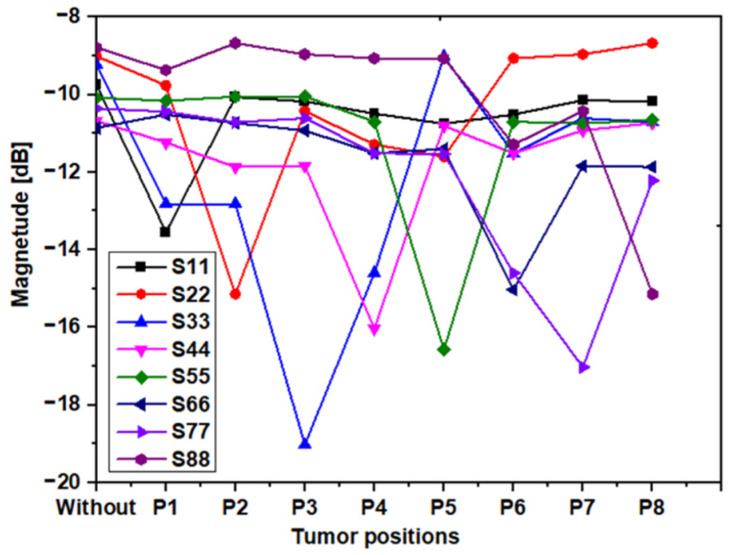
Experimental results of S_ii_ in a different position at 5.34 GHz.

In our experiment, we placed the tumor in different locations inside the breast phantom and measured the Sii parameters. Figure 18 exemplifies the output of our tests for the one tumor position. Figure 19 illustrates the measured results of S_ii_ versus the tumor position at 5.34 GHz.

It is possible to notice from Figure 18 that each antenna’s return loss is lowest when the tumor is closest to it (S_11_ is lowest for P1, S_22_ for P2 … and S_88_ for P8). Therefore, the proposed system is a good candidate not only for determining tumor detection but also for tumor position. As shown in Figure 19, if S_11_ has a minimum value, then we can conclude that there is a tumor in position 1, as is the case for the other antennas. Additionally, when there is no peak in the S_ii_ values of the eight antennas, then there is no tumor and, therefore, the breast is not infected.

## 4. Conclusions

This paper presented the conception and design of a sensing antenna for breast cancer detection. The proposed array of Vivaldi-like antennas is useful as a sensor for tumor detection because of its relatively high radiation efficiency, flexibility and small size. In this paper, a Vivaldi antenna dedicated to tumor detection, with a directive radiation pattern for more accuracy, was designed and optimized, arriving at a reduction from 77.66 × 63.35 mm^2^ to 67 × 46 mm^2^ (a 33% decrease), without gain degradation. The directional radiation property (narrow lobe) of this antenna not only ensures that enough energy enters the phantom but also reduces the illumination area, which increases the resolution of the microwave measurements. The experimental results are consistent with those obtained in the simulation.

## Figures and Tables

**Table 1:** Mechanical parameters of the basic Vivaldi antenna (all parameters are presented in mm).

Parameters	L	L1	L2	W	W1	Arc1	Arc2
Values (mm)	77.66	12.68	34.35	63.35	1.51	Equation (1)	Equation (2)

**Table 2:** Mechanical parameters of the modified Vivaldi antenna based on the MTM cell.

Parameters	L	L1	L2	W	W1	W2	Arc1	Arc2
Values (mm)	67	12.68	19.78	46	1.51	10.74	Equation (1)	Equation (2)

**Table 3:** Comparison study of the proposed work with some existing antenna used for breast cancer detection.

Antenna Types	Operating Frequency Range (GHz)	Quality Factor: Q	RL (dB)	Gain [dB]	Size [mm^3^]	Front to Back Ratio	Fabrication Complexity
Microstrip antenna [14]; 2018	(1.6, 11.2)	0.67	20	4.02	70 × 60 × 1.6	(−−)	(−−+)
CPW antenna [15]; 2021	(1.85, 8.37)	1.27	15	3.12	43 × 43 × 1.6	(−−)	(−−−)
Metamaterial CPW antenna [4]; 2022	(2.42–3.3) (4–15)	3.25 1.158	18 25	2 6	80 × 61 × 6	(−−+)	(+)
Multilayered antenna (3D) [16]; 2021	(4.8, 30)	0.69	20	--	24 × 24 × 1.4	(−+)	(++)
Monopole antenna [17]; 2020	(3.22, 11.92)	0.87	20	6	30 × 29 × 1.6	(−−)	(−−−)
Elliptical UWB antenna with MTM arrays [18]; 2020	(6.5, 35)	0.728	26	8.85	10 × 15 × 2.2	(−+)	(++)
Cubical-shaped dielectric resonator antenna [7]; 2021	(4.3, 12.6)	1.01	32	6	20 × 15 × 5.6	(−−)	(+)
Vivaldi antenna [19]; 2016	(0.8, 1.2) (2.1, 2.5)	2.5 5.75	14 13		32.4 × 13.5 Box: 60 × 60 × 30	(++)	(−)
Metamaterial Vivaldi antenna [This paper]	(4.6, 8)	1.85	26	5.8	67 × 46 × 0.64	(+++)	(−−)

## Data Availability

The data presented in this study are available on request from the corresponding author.
